# Training calibration-based counterfactual explainers for deep learning models in medical image analysis

**DOI:** 10.1038/s41598-021-04529-5

**Published:** 2022-01-12

**Authors:** Jayaraman J. Thiagarajan, Kowshik Thopalli, Deepta Rajan, Pavan Turaga

**Affiliations:** 1 Lawrence Livermore National Labs, Livermore, 94550 USA; 2 Geometric Media Lab, Arizona State University, Tempe, 85281 USA; 3 Milpitas, 95035 USA

**Keywords:** Diagnosis, Radiography

## Abstract

The rapid adoption of artificial intelligence methods in healthcare is coupled with the critical need for techniques to rigorously introspect models and thereby ensure that they behave reliably. This has led to the design of explainable AI techniques that uncover the relationships between discernible data signatures and model predictions. In this context, counterfactual explanations that synthesize small, interpretable changes to a given query while producing desired changes in model predictions have become popular. This under-constrained, inverse problem is vulnerable to introducing irrelevant feature manipulations, particularly when the model’s predictions are not well-calibrated. Hence, in this paper, we propose the TraCE (training calibration-based explainers) technique, which utilizes a novel uncertainty-based interval calibration strategy for reliably synthesizing counterfactuals. Given the wide-spread adoption of machine-learned solutions in radiology, our study focuses on deep models used for identifying anomalies in chest X-ray images. Using rigorous empirical studies, we demonstrate the superiority of TraCE explanations over several state-of-the-art baseline approaches, in terms of several widely adopted evaluation metrics. Our findings show that TraCE can be used to obtain a holistic understanding of deep models by enabling progressive exploration of decision boundaries, to detect shortcuts, and to infer relationships between patient attributes and disease severity.

## Introduction

There is a growing interest in adopting artificial intelligence (AI) methods for critical decision-making, from diagnosing diseases to prescribing treatments and allocating resources, in healthcare^1–3^. However, in order to trust AI systems and to prioritize patient safety, it is imperative to ensure those methods are both accurate and reliable^4^. Examples of unreliable AI systems include a model that can produce highly confident predictions for patients presenting anomalies not seen in the training data, or a model accumulating evidence for a certain diagnosis based on uninformative regions in an image^5,6^. This has strongly motivated the need to both reliably assess a model’s confidence in its predictions^7–9^, and to enable rigorous introspection of its behavior^4,10–12^. To this end, uncertainty estimation methods are being adopted to determine the deficiencies of a model and/or the training data^13^. Meaningful uncertainties can play a crucial role in supporting practical objectives that range from assessing regimes of over (or under)-confidence and active data collection, to ultimately improving the predictive models themselves^14^. However, in practice, uncertainties are known to be challenging to communicate to decision-makers^15^, and the robustness of decisions with respect to uncertainties can vary considerably between use-cases^16^. Consequently, it could sometimes be more beneficial to implicitly leverage uncertainties when performing introspective analysis of machine-learned models.

Model introspection approaches that attempt to explain the input-output relationships inferred by models are routinely used to understand and promote trust in AI solutions. While there has been a large body of work on building inherently explainable models (e.g., rule based systems), post-hoc explanation methods have become the modus operandi with modern deep learning systems^17^. In particular, *local* explanation methods are very popular as they provide a convenient way for users to introspect by generating local explanations specific to a given input (e.g., health record of a patient)—elucidate what features in the input data maximally support the prediction. Broadly, local explanation methods can be categorized into approximation and example-based approaches. The former class of methods begin by sampling in the vicinity of a query example and fit an explainer to the chosen set of samples (e.g., fit a linear model in LIME^18^ or extract rules in ANCHORS^19^). In contrast, example-based methods synthesize data samples in the vicinity of a query, such that the predictions for those samples align with a user-specified hypothesis. The data samples from the latter approach are referred to as *counterfactual* explanations^20,21^. While counterfactual explanations provide more flexibility over feature importance estimation methods, user-studies have also demonstrated that counterfactuals can elicit meaningful insights into the data^22^.

While it is common to utilize counterfactuals for causal reasoning, in the recent years, they have been found to be effective for scenario exploration even with predictive models^23,24^. In its most generic form, for a given query ${\text {x}}$, one can pose counterfactual generation based on a predictive model ${\text {F}}: X \rightarrow Y$ as an optimization problem:

1 $$\begin{aligned} \arg \min _{\bar{{\text {x}}}} d({\text {x}},\bar{{\text {x}}}) \quad {\text{ s.t. }} \quad {\text {F}}(\bar{\text {x}}) = \bar{\text {y}}; \text { } \bar{{\text {x}}} \in M({X}) \end{aligned}$$

where $\bar{{\text {x}}}$ is a counterfactual explanation for the query $\text {x}$ (e.g., a medical image of a patient) and $\bar{{\text {y}}}$ is the user-specified hypothesis about $\bar{{\text {x}}}$ (e.g., a certain diagnosis). Minimizing a suitable discrepancy *d*(., .) between ${\text {x}}$ and $\bar{{\text {x}}}$ ensures that the underlying semantic content of $\text {x}$ is preserved in the counterfactual (i.e., vicinity). Another important requirement to produce meaningful counterfactuals is that the generated $\bar{{\text {x}}}$ should lie close to the original data manifold *M*(*X*). When no tractable priors exist for *M*(*X*), it is common to perform this optimization in the latent space of a pre-trained generative model (e.g., variational autoencoders (VAE)^25^ or generative adversarial networks (GAN)^26^). Despite the effectiveness of such priors, when the model’s predictions ${\text {F}}(\bar{{\text {x}}})$ are poorly calibrated, i.e., prediction confidences are not indicative of the actual likelihood of correctness^9,27^, the optimization in Eq. (1) can still lead to bad quality explanations. Though different variants of the formulation in Eq. (1) have been considered in the literature^20^, the fundamental challenge with uncalibrated predictions still persists. We propose to circumvent this challenge by integrating prediction uncertainties into the counterfactual generation process.

### Proposed work

**Figure 1:**
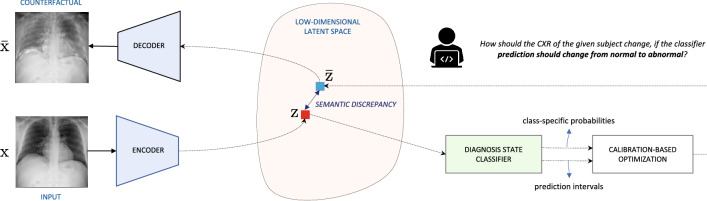
An overview of TraCE applied for introspective analysis of chest X-ray (CXR)-based predictive models. In this example, we consider a binary classifier that has been trained to distinguish between *normal* and *abnormal* subjects (i.e., containing pneumonia-related anomalies). Since TraCE carries out the optimization in the latent space of a pre-trained auto-encoder model, we first transform a query image ${\text {x}}$ (from the *normal* class) into its latent representation $\text {Z}$ using the *Encoder*. Subsequently, we invoke the proposed calibration-driven optimization to obtain the counterfactual $\bar{{\text {x}}}$ in the latent space, such that the semantic discrepancy between ${\text {z}}$ and $\bar{{\text {z}}}$ is minimized and the classifier’s prediction changes to *abnormal*. Note that, the classifier is trained to output the probabilities for each of the classes along with the prediction intervals. Finally, the synthesized counterfactual $\bar{{\text {z}}}$ is transformed into the image-space ($\bar{{\text {x}}}$) using the *Decoder* network.

In this work, we propose TraCE (*Training Calibration-based Explainers*), an introspection method for deep medical imaging models, that effectively leverages uncertainties to generate meaningful counterfactual explanations for clinical image predictors. As illustrated in Fig. 1, our framework is comprised of three key components: (1) an auto-encoding convolutional neural network to construct a low-dimensional, continuous latent space for the training data; (2) a predictive model that takes as input the latent representations and outputs the desired target attribute (e.g., diagnosis state, age etc.) along with its prediction uncertainty; and (3) a counterfactual optimization strategy that uses an uncertainty-based calibration objective to reliably elucidate the intricate relationships between image signatures and the target attribute. While our approach is flexible to support the use of any uncertainty estimator or prediction models that use explicit regularization to produce well-calibrated predictions, TraCE builds upon the recent Learn-by-Calibrating (LbC) technique^28^ to obtain prediction intervals for both classification and regression settings. LbC jointly trains an auxiliary interval estimator alongside the predictor model using an interval calibration objective, and has been shown to be effective at recovering complex function mappings in scientific datasets. We first adapt LbC for multi-class classification problems and subsequently propose a counterfactual generation approach based on the estimated prediction intervals. When compared to interpretability techniques that provide saliency maps or feature importance scores to explain a specific decision^29^, TraCE enables progressive transition between different output states (e.g., *normal* $\rightarrow$ *abnormal*) through appropriate image manipulations and more importantly, allows optimization with both categorical- and continuous-valued target variables.

Our key contributions can be summarized as follows: (1) a new calibration-based optimization approach that takes into account prediction uncertainties; (2) a generalized version of the recent Learn-by-Calibrating technique for classification settings; (3) novel analysis based on TraCE for progressive exploration of decision boundaries, detecting shortcuts and inferring attribute relationships; and (4) empirical studies with a CXR-based modeling use-case and rigorous evaluation with respect to existing baselines.

### Results

While recent advances in ML such as deep learning have produced disruptive innovations in many fields, radiology is a prominent example. Conventionally, trained physicians visually assess medical images for characterization and monitoring of diseases. However, AI methods have been showed to be effective at automatically identifying complex signatures in imaging data and providing quantitative assessments of radiographic characteristics^30^. Motivated by this wide-spread adoption of AI tools in radiology^31–33^, our study focuses on detecting anomalies in chest X-ray (CXR) images. More specifically, we use images from the publicly available RSNA pneumonia detection challenge database (https://www.kaggle.com/c/rsna-pneumonia-detection-challenge) in order to demonstrate the effectiveness of TraCE in performing introspective analysis. Given the diagnosis task of categorizing Chest X-ray (CXR) images into *normal* and *abnormal* groups (i.e., pneumonia-related anomalies), one can adopt a variety of ML solutions including deep neural networks to build classifiers. However, in practice, purely data-driven AI solutions can learn unintended *shortcuts*^34^ (e.g., superficial correlations) instead of meaningful decision rules. Such models typically perform well on the observed data, including passing standard cross-validation tests, yet fail when deployed in the real-world^5,6^. Hence, the foremost utility of TraCE is in validating that a predictive model has learned generalizable decision rules.

To this end, we use TraCE to progressively generate counterfactuals with different levels of likelihood in assigning a patient to the *abnormal* group. From our results, we find that using TraCE consistently produces highly meaningful counterfactual evidences, wherein severity of *abnormality* (e.g., pneumonia) is characterized primarily by changes in the lung opacity. More importantly, the image manipulations are highly concentrated in the chest region of the subject, thus showing that the models did not pick shortcut inductive biases (e.g., scanner-specific features or background image pixels). Using rigorous empirical studies, we also show that TraCE outperforms existing baseline methods, in terms of several widely adopted evaluation metrics in counterfactual reasoning. Furthermore, we find that TraCE can effectively detect shortcuts (or unintended biases) in trained models and infer relationships between different attributes (for example, age and diagnosis state), thus enabling a holistic understanding of deep clinical models.

## Background and related work

### Uncertainty estimation

The growing interest in employing machine learning (ML) based solutions to design diagnostic tools and to gain new insights into a host of medical conditions strongly emphasizes the need for a rigorous characterization of ML algorithms. In conventional statistics, uncertainty quantification (UQ) provides this characterization by studying the impact of different error sources on the prediction^35–37^. Consequently, several recent efforts have proposed to utilize prediction uncertainties in deep models to shed light onto when and how much to trust the predictions^38–40^. Some of the most popular uncertainty estimation methods today include: (1) Bayesian neural networks^37,41^: (2) methods that use the discrepancy between different models as a proxy for uncertainty, such as deep ensembles^42^ and Monte–Carlo dropout that approximates Bayesian posteriors on the weight-space of a model^38^; and (3) approaches that use a single model to estimate uncertainties, such as orthonormal certificates^43^, deterministic uncertainty quantification^44^, distance awareness^45^, depth uncertainty^46^, direct epistemic uncertainty prediction^47^ and accuracy versus uncertainty calibration^48^.

### Prediction calibration

It has been reported in several studies that deep predictive models need not be inherently well-calibrated^27^, i.e., the confidences of a model in its predictions are not correlated to its accuracy. While uncertainties can be directly leveraged for a variety of downstream tasks including out-of-distribution detection and sequential sample selection, they have also been utilized for guiding models to produce well-calibrated predictions. In practice, these requirements are incorporated as regularization strategies to systematically adjust the predictions during training, most often leading to better performing models. For example, uncertainties from Monte–Carlo dropout^49^ and direct error prediction^50^ have been used to perform confidence calibration in deep classifiers. Similarly, the recently proposed Learn-by-Calibrating (LbC) approach^28^ introduced an interval calibration objective based on uncertainty estimates for training deep regression models.

### Counterfactual generation in predictive models

Counterfactual (CF) explanations^20^ that synthesize small, interpretable changes to a given image while producing desired changes in model predictions to support user-specified hypotheses (e.g., progressive change in predictions) have recently become popular. An important requirement to produce meaningful counterfactuals is to produce discernible local perturbations (for easy interpretability) while being realistic (close to the underlying data manifold). Consequently, existing approaches rely extensively on pre-trained generative models to synthesize plausible counterfactuals^20,21,51–53^. While the proposed TraCE framework also utilizes a pre-trained generative model, it fundamentally differs from existing approaches by employing uncertainty-based calibration for counterfactual optimization.

## Results

### Data

Our analysis uses CXR images available as public benchmark data for the tasks of predicting the diagnostic state and other patient attributes. In particular, our study uses the *RSNA pneumonia detection challenge database*, which is a collection of 30,000 CXR exams belonging to the NIH CXR14 benchmark dataset^54^, of which 15,000 exams show evidence for lung opacities related to pneumonia, consolidation and infiltration, and 7500 exams contain no findings (referred as *normal*). The CXR images in the dataset were annotated by six board-certified radiologists and additional information on the data curation process can be found in Ref.^55^. In addition to the diagnostic labels, this dataset contains age and gender information of the subjects. Note that, for this analysis, we used healthy control subjects from the RSNA pneumonia dataset to define the *normal* group and designed predictive models to discriminate them from patients presenting pneumonia-related anomalies in their CXR scans. We refer to the latter as the *abnormal* group.

### Evaluation metrics

We used the following metrics for a holistic evaluation of the counterfactual explanations obtained using TraCE and other baseline methods.

1. *Validity* For categorical attributes (as in classification problems), this metric measures the ratio of the counterfactuals that actually have the desired target attribute to the total number of counterfactuals generated (higher the better). In the case of continuous-valued attributes we measure the mean absolute percentage error (MAPE) between the desired and achieved target values (lower the better).
  2 $$\begin{aligned} V_{cat}&= \frac{1}{N}\sum _{i=1}^N {\mathbb {I}}({\text {F}}({\text {x}}_i), \bar{{\text {y}}}_i); \quad V_{cont} = \frac{1}{N}\sum _{i=1}^N \left| \frac{\bar{{\text {y}}}_i - {\text {F}}({\text {x}}_i)}{\bar{{\text {y}}}_i}\right| , \end{aligned}$$
  where ${\mathbb {I}}$ denotes the identity function that returns 1 when the arguments match, and *N* is the total number of query samples used for evaluation.
2. *Confidence* In cases of categorical-valued targets (class labels), we compute the confidence $P(\bar{{\text {y}}}_i|\bar{{\text {x}}}_i; {\text {F}})$ (from softmax probabilities) of assigning the desired class $\bar{{\text {y}}}_i$ for a counterfactual $\bar{{\text {x}}}_i$ (higher the better).
3. *Sparsity* Since we perform optimization directly in the latent space, measuring the amount of change in the images is a popular metric in the literature. We compute the sparsity metric as the ratio of the number of pixels altered to the total number of pixels.
  3 $$\begin{aligned} S({\text {x}}) = \frac{\sum _i \sum _j C(|{\text {x}}_{ij} - \bar{{\text {x}}}_{ij}|)}{T}, {\text { where }} C(a) = {\left\{ \begin{array}{ll} 1, &{}\quad {\text {if }} a > 0\\ 0, &{}\quad {\text {otherwise}}, \end{array}\right. } \end{aligned}$$
  and *T* denotes the total number of pixels in the query ${\text {x}}$. In general, sparser changes to an image are more likely to preserve the inherent characteristics of the query image.
4. *Proximity* Recent works have considered the actionability of modified features by grounding them in the training data distribution. Following^56^, we measure the average $\ell _2$ distance of each counterfactual to the K-nearest training samples in the latent space (lower the better)
  4 $$\begin{aligned} Prox(\bar{{\text {x}}}) = \frac{1}{K} \sum _{{\text {x}} \in {\text {N}}_K(\bar{{\text {x}}}; X)} \Vert {\text {E}}(\bar{\text {x}}) - {\text {E}}({\text {x}})\Vert _2, \end{aligned}$$
  where ${\text {N}}_K(\bar{{\text {x}}}; X)$ denotes the set of *K* nearest neighbors of the counterfactual ${\text {x}}$ from the training data *X*, and ${\text {E}}$ denotes the encoder network (see “Methods” section for details) to compute the latent representation.
5. *Realism score* We also employ this metric from the generative modeling literature^57^ to evaluate the quality of images obtained using TraCE. While standard metrics such as the FID (Frechét Inception Distance) score or the precision/recall metrics are used to evaluate the overall quality of a population of generated images, they are not sufficient to assess individual images. Hence, we utilize the realism score introduced in Ref.^58^, which is high when the generated image is close to the true data manifold and decreases as the image moves further from the manifold. Denoting the feature vectors for the set of real images (used for training), obtained using a pre-trained classifier such as VGG-16, by the matrix $\varvec{\Psi }_r = [\psi _r^1, \ldots , \psi _r^N]$ and a generated image by $\psi _g$, the realism score can be computed as follows:
  5 $$\begin{aligned} R(\psi _g, \varvec{\Psi }_r) = \max _{\psi _r^j} \left\{ \frac{\Vert \psi _r^j - \psi _r^K \Vert _2}{\Vert \psi _r^j - \psi _g\Vert _2}\right\} , \end{aligned}$$
  where $\psi _r^K$ refers to the feature vector corresponding to the $K{\text {th}}$ nearest neighbor (w.r.t to $\varvec{\Psi }_r$) from the set $N_K(\psi _r^j; \varvec{\Psi }_r)$.

### TraCE enables progressive exploration of decision boundaries

Given the rapid adoption of AI solutions in diagnosis and prognosis, it is critical to gain insights into black-box predictive models. In this study, we analyzed a predictive model that classifies CXR images into *normal* and *abnormal* groups, and used TraCE to synthesize counterfactuals for a given query image from the *normal* class to visualize the progression of disease severity. Such an analysis can reveal what image signatures are introduced by a predictive model to provide evidence for the *abnormal* class, and can be used by practitioners to verify if the model relies on meaningful decision rules or *shortcuts* (e.g., changes to the background) that cannot generalize. In our implementation of TraCE, we first constructed a low-dimensional latent space (100 dimensions) for the dataset of CXR images using a Wasserstein auto-encoder^59^. We subsequently learned the predictive model ${\text {F}}_D$ along with the interval estimator ${\text {G}}_D$, using a modified version of the LbC algorithm (details in the “Methods” section). The hyper-parameters $\eta _1$ and $\eta _2$ in Eq. (11) are critical to trade-off between preserving the inherent semantics from query ${\text {x}}$ and achieving the desired prediction. Hence, one can progressively transition from the *normal* to the *abnormal* class by fixing $\eta _2$ and gradually relaxing $\eta _1$.

**Figure 2:**
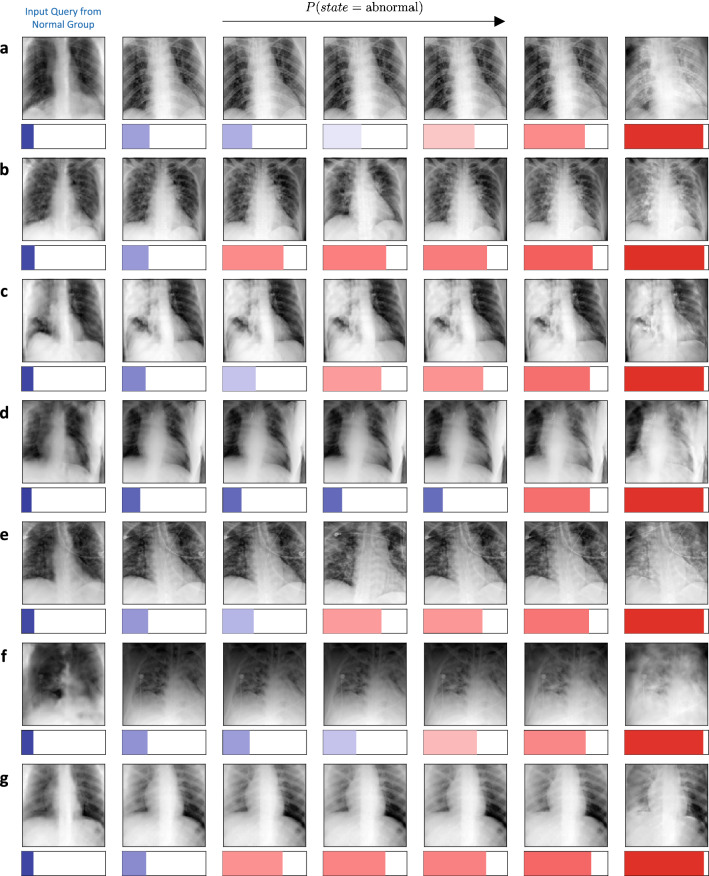

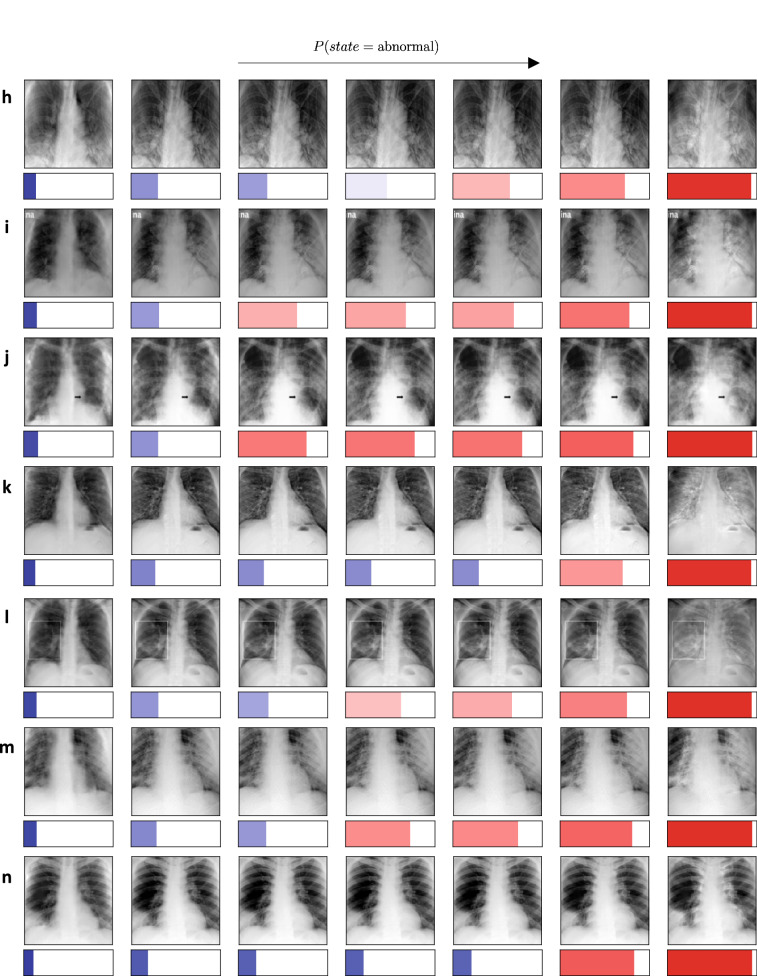
(**a**–**g**) Diagnosis-based counterfactual explanations generated using TraCE by progressively introducing relevant patterns into different query images (first image in each row) of healthy subjects to increase the likelihood of being assigned to the *abnormal* group ($P(state = {{abnormal}})$). (**h**–**n**) Diagnosis-based counterfactual explanations generated using TraCE by progressively introducing relevant patterns into different query images (first image in each row) of healthy subjects to increase the likelihood of being assigned to the *abnormal* group ($P(state = {{abnormal}})$).

Figure 2 illustrates the counterfactuals obtained using TraCE for multiple different examples from our benchmark dataset. More specifically, the query samples $\text {x}$ correspond to CXR images from the *normal* class and we varied $\eta _1$ between 0.5 and 0.05, while setting $\eta _2 = 0.5$ and $\eta _3 = 0.2$. These values were obtained using a standard hyper-parameter search based on 500 randomly chosen images. For each case from Fig. 2, the different counterfactuals along with their estimated $P(state = {{abnormal}})$ from the predictive model ${\text {F}}_D$ are shown. It can be clearly observed from the results that the counterfactuals show increased opacity in the lung regions (appearing as denser white clouds) as we progress towards the *abnormal* class, which strongly corroborates with existing studies on CXR-based image analysis. Furthermore, TraCE does not arbitrarily introduce irrelevant features into the image or make anatomical changes, thereby reliably preserving the inherent characteristics of the subject. By producing physically plausible evidences for crucial hypotheses, TraCE enables practitioners to effectively explore complex decision boundaries learned by deep predictive models.

### Comparing TraCE to baseline methods

In order to perform a quantitative evaluation of TraCE, we obtained counterfactuals for 500 randomly chosen images from a held-out test set (not used for training) and Table 1 presents a detailed comparison of different baseline methods (see “Methods” section for details). Note that, we chose the hyper-parameters $\eta _1, \eta _2, \eta _3$ such that the average discrepancy in the latent space is similar across all methods. The first striking observation is that, despite using the same pre-trained latent space for counterfactual optimization, all methods that incorporate explicit calibration strategies or uncertainty estimation consistently outperform the *Vanilla* model. More specifically, for similar levels of discrepancy in the latent space, TraCE achieves a significantly higher validity score of 0.88 as opposed to 0.69 of the *Vanilla* model, while inducing similar or lower amount of changes to the query (indicated by the sparsity and proximity metrics). Furthermore, our approach outperforms the results obtained with state-of-the-art uncertainty estimators and calibration strategies (in all the metrics), thus demonstrating its efficacy in generating counterfactual explanations.

**Table 1:** Performance evaluation of diagnosis-based counterfactual explanations obtained using different approaches. In each case, we report results averaged across 500 test samples.

Method	Validity $\uparrow$	Confidence $\uparrow$	Sparsity $\downarrow$	Proximity $\downarrow$	Realism $\uparrow$
Vanilla	0.68	0.63±0.11	0.3±0.17	4.59±0.68	1.16 ± 0.09
Mixup	0.78	0.69±0.17	0.27±0.16	4.09±0.52	1.19 ± 0.13
UWCC	0.79	0.75±0.13	0.25±0.17	4.26±0.63	1.16 ± 0.2
MC dropout	0.73	0.66±0.16	0.34±0.19	4.57±0.53	1.18 ± 0.16
Deep ensembles (5 models)	0.8	0.72±0.09	0.29±0.11	**3.68**±**0.57**	1.21 ± 0.12
TraCE	**0.87**	**0.81**±**0.12**	**0.23**±**0.14**	3.73±0.51	**1.33** ± **0.13**

Significant values are in [bold].

As discussed earlier, TraCE is applicable for predictive models outputting both categorical- and continuous-valued target variables. To demonstrate this, we considered only healthy control subjects from the RSNA dataset and designed a regressor to estimate their age attribute using their CXR images. Though the age prediction task is not necessarily relevant on its own in clinical diagnosis, as we will show next, such attribute estimators can be utilized for inferring relationships to the diagnosis state. For our evaluation, we used 500 randomly chosen test subjects whose age attribute was between 40 and 70 and set the desired value $\bar{{\text {y}}} = 20$. From Table 2, we notice that the proposed approach achieves lower validity (MAPE) scores, without compromising on the proximity metric, when compared to the other baselines. Interestingly, we find that changing the age attribute required the manipulation of much lesser number of pixels (low sparsity values) when compared to the diagnosis state.

**Table 2:** Performance evaluation of age-based counterfactual explanations obtained using different approaches. In each case, we report results averaged across 500 test samples.

Method	Validity $\downarrow$	Sparsity $\downarrow$	Proximity $\downarrow$	Realism $\uparrow$
Vanilla	2.49	0.06±0.08	4.08±0.48	1.26±0.1
Mixup	0.83	**0.05**±**0.07**	3.79±0.52	1.28±0.07
UWCC	0.74	0.09±0.03	3.81±0.42	1.33±0.05
MC dropout	1.44	0.07±0.08	4.13±0.29	1.26±0.06
Deep ensembles (5 models)	0.45	**0.05**±**0.09**	3.89±0.32	1.32±0.06
TraCE	**0.16**	**0.05**±**0.03**	**3.66**±**0.35**	**1.38** ± **0.06**

Significant values are in [bold].

### TraCE detects shortcuts in deep models

**Figure 3:**
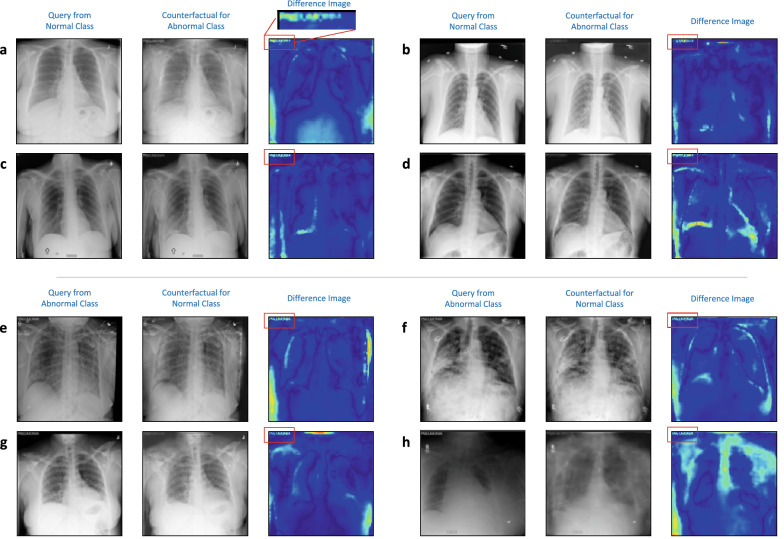
Using TraCE to detect shortcuts in deep predictive models. In this experiment, we synthetically introduced a nuisance feature (overlaid the text PNEUMONIA in the top-left corner) into all images from the *abnormal* group, and used this data to train the predictive model. Given the entirely data-driven nature of machine-learned solutions, there is risk of inferring a decision rule based on this irrelevant feature in order to discriminate between *normal* and *abnormal* groups. (**a**–**d**) Here, we used randomly chosen query images from the *normal* class and generated counterfactuals for the *abnormal* class. In each case, we show the query image, the counterfactual explanation from TraCE and the absolute difference image between the two; (**e**, **f**) Here, we introduced the nuisance feature into CXR images from the *abnormal* group and synthesized counterfactuals for the *normal* class. We observe that TraCE can effectively detect such shortcuts—counterfactuals for changing the diagnosis state are predominantly based on manipulating the text on the top-left corner of the query images.

An important challenge with purely data-driven methods is that they have the risk of inferring decision rules based on shortcuts, thereby limiting their utility in practice. Detecting such shortcuts is essential to both validate model behavior and to detect unintended biases (hospital-specific or device-specific information) in the training data. In order to demonstrate the use of TraCE in detecting such shortcuts, we synthetically introduced a *nuisance* feature into images from the *abnormal* class—overlaid the text PNEUMONIA in the top-left corner of each image, and used TraCE to check if the model’s decision was based on this nuisance feature. After training the Wasserstein autoencoder and the LbC model using the altered images, we selected query images from the *normal* group and generated the corresponding counterfactual evidences for the *abnormal* group. As illustrated in Fig. 3a–d, TraCE exclusively manipulates the top-left corner to accumulate evidence for abnormality, thus revealing that the predictive model relies on the nuisance feature. Similarly, in Fig. 3e–h, one can transition from the *abnormal* (examples containing the nuisance feature) to the *normal* group by simply removing the synthetic text PNEUMONIA. This experiment clearly emphasizes the utility of TraCE in detecting model and data biases.

### TraCE reveals attribute relationships

Motivated by the effectiveness of TraCE in producing counterfactuals for different types of target attributes, we next explored how counterfactual optimization can be used to study relationships between patient attributes, such as age and gender, and the diagnosis state. Note, this analysis is based on the assumption that the patient attribute can be directly estimated from the CXR images, and the inferred relationship does not necessarily imply causality.

First, we study if the image signatures pertinent to the patient age attribute provides additional evidence for diagnosis state prediction. Given the age predictor, along with its interval estimator, $({\text {F}}_A, {\text {G}}_A)$ and the diagnosis predictor $({\text {F}}_D, {\text {G}}_D)$, we constructed counterfactuals based on two independent hypotheses. Note, both predictors were constructed based on the same low-dimensional latent representations. More specifically, we provided the hypotheses ${\bar{y}}_A = 70$ and ${\bar{y}}_D = {{abnormal}}$ for the two cases, and used TraCE to generate counterfactuals $\bar{{\text {x}}}_A$ and $\bar{{\text {x}}}_D$ that adhere to our hypotheses. We then estimated the age-specific and diagnosis-specific signatures introduced by TraCE:

6 $$\begin{aligned} \Delta _A({\text {x}}) = {\text {x}} - \bar{{\text {x}}}_A; \quad \Delta _D({\text {x}}) = {\text {x}} - \bar{{\text {x}}}_D. \end{aligned}$$

In order to check if there exists an apparent relationship between age and diagnosis state, we generated the hybrid counterfactual,

7 $$\begin{aligned} \bar{{\text {x}}} = {\text {x}} + \Delta _A({\text {x}}) + \Delta _D({\text {x}}). \end{aligned}$$

**Figure 4:**
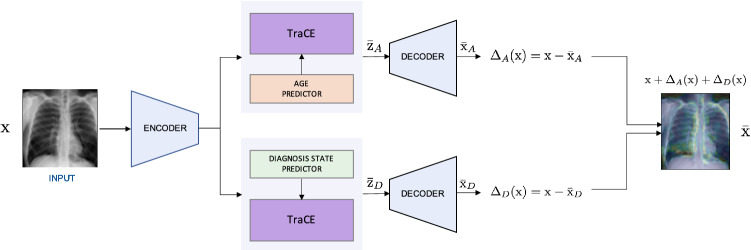
Using TraCE to infer relationships between a patient attribute (e.g., age) and disease states. For this analysis, we construct two independent predictive models, i.e., age and diagnosis state, and synthesize counterfactuals based on hypothesis on each of the predictions (e.g., patient age should be predicted as 70 while the diagnosis state should be *abnormal*. Finally, we combine the changes induced in the two counterfactuals, $\Delta _A({\text {x}})$ and $\Delta _D({\text {x}})$ respectively, and check if incorporating age-specific patterns strengthens the evidence for the *abnormal* class.

Finally, we compared ${\text {F}}_D(\bar{{\text {x}}}) - {\text {F}}_D(\bar{{\text {x}}}_D)$ to quantify if incorporating age-specific features into $\bar{{\text {x}}}_D$ increased the disease severity (i.e., likelihood of being assigned to the *abnormal* class). An overview of this strategy is illustrated in Fig. 4.

**Figure 5:**
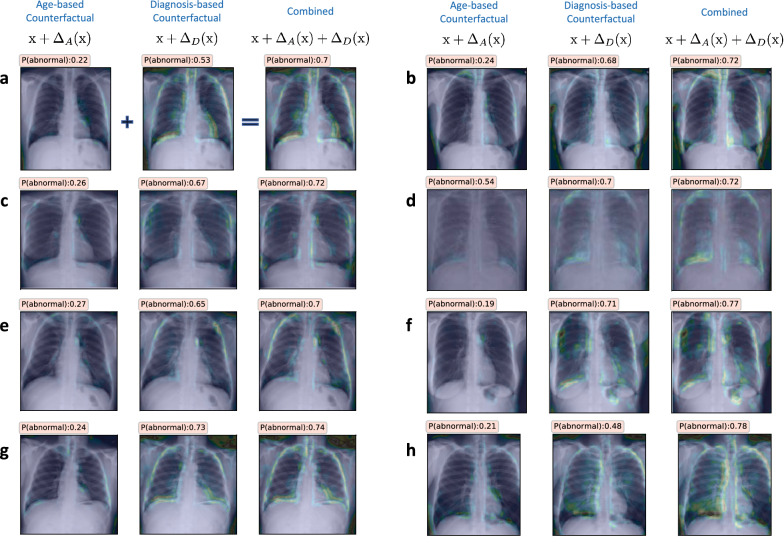
(**a**–**h**) Explanations generated using TraCE by introducing age-specific attributes into the counterfactuals synthesized for changing the diagnosis state of a *normal* subject to be *abnormal*. Interestingly, we find that there exists a correlation between the two attributes, as evidenced by the consistent increase in the likelihood $P(state = {{abnormal}})$ when compared to counterfactuals that rely only on patterns from the diagnosis state predictor. In each case, we highlight the changes $\Delta _A({\text {x}}), \Delta _D({\text {x}}),(\Delta _A({\text {x}})+\Delta _D({\text {x}}))$ and display the likelihood $P(state = {{abnormal}})$.

**Figure 6:**
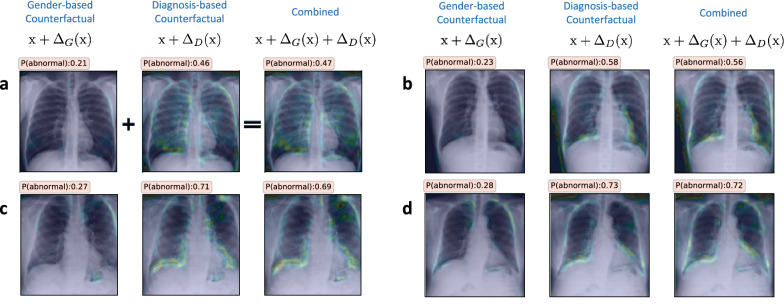
(**a**–**d**) Explanations generated using TraCE by introducing gender-specific attributes into the counterfactuals synthesized for changing the diagnosis state of a *normal* subject to be *abnormal*. In contrast to the age attribute, image manipulations associated with change in gender (female $\rightarrow$ male) do not cause any apparent change to the likelihood of being assigned to the *abnormal* group. In each case, we highlight the changes $\Delta _A({\text {x}}), \Delta _D({\text {x}}),\Delta _A({\text {x}})+\Delta _D({\text {x}})$ and show the likelihood $P(state = {{abnormal}})$.

Figure 5 shows the results for eight different *normal* subjects, wherein we find that there is an apparent increase in $P(state={{abnormal}})$ when age-specific signatures are incorporated. Using 500 randomly chosen *normal* subjects, we estimated an average change of $0.09 \pm 0.08$ in ${\text {F}}_D(\bar{{\text {x}}}) - {\text {F}}_D(\bar{{\text {x}}}_D)$, thus indicating that the diagnosis predictor is sensitive to age-specific patterns. In practice, if such a relationship is expected, it is a strong validation for the model’s behavior. On the other hand, if the attribute is a confounding variable, it becomes critical to retrain the model wherein this sensitivity is explicitly discouraged. Interestingly, when we repeated this analysis with the gender attribute, such a relationship was not apparent (see results in Fig. 6).

## Methods

In this section, we discuss in detail the methodology for performing calibration-driven counterfactual generation. All the methods presented were performed in accordance with the relevant guidelines and regulations.

### Constructing low-dimensional latent spaces

**Figure 7:**
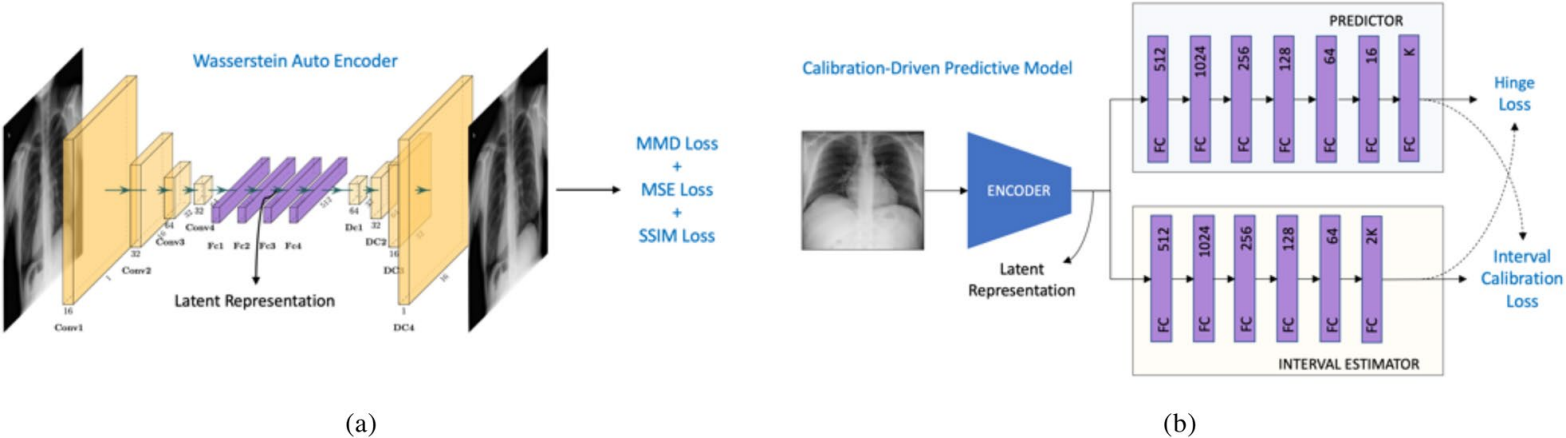
Framework design for TraCE. (**a**) First, we train an auto-encoding neural network^59^, and construct a low-dimensional, continuous latent space for CXR images. Note, we used a combination of maximum mean discrepancy (MMD), mean squared error (MSE) and structural similarity (SSIM) losses to train the network parameters; (**b**) next, we adapt the Learn-by-Calibrating^62^ approach to train a classifier that takes as input the latent representation from the encoder and outputs a patient-specific attribute along with prediction intervals.

Given a set of samples from an unknown data distribution, our goal is to build a low-dimensional, continuous latent space that respects the true distribution, so that one can generate counterfactual representations in that space. A large class of generative modeling methods exist to construct such a latent space. In this work, we focus on Wasserstein autoencoders^59^ since they have been found to outperform other variational autoencoder formulations, particularly in image datasets with low heterogeneity, e.g., scientific images from physics simulations^60^. This network is a composition of an encoder network $\text {E}$ that transforms the input $\text {x}$ into its latent code $\text {z}$, and a decoder network $\text {D}$ that reconstructs the image. Additionally the encoder has the objective of matching the latent distribution of the training samples ${\mathbb {E}}_{P_{X}}[{\text {E}}({\text {z}} \mid {\text {x}})]$ to a pre-specified prior $P_{Z}$. This helps us to sample from the prior as well as generate new unseen samples from the original data manifold *M*(*X*) after training such auto-encoding models. Wasserstein autoencoders thus have to minimize: (1) discrepancy cost $D_x$ between the original data distribution and the generated; (2) discrepancy cost $D_z$ between the latent distribution of the encoded training samples to that of a prior. Following standard WAE consturction, we employ the mean squared error (MSE) for $D_x$ and use the maximum mean discrepancy (MMD) to define $D_z$. As shown in Fig. 7a, we also find that including another loss term to maximize the structural similarity (SSIM)^61^ between the original and reconstructed images led to higher quality reconstructions.

#### Training

All images were resized to $224\times 224$ pixels, and treated as single channel images. With the latent space dimensionality fixed at 100, the encoder model was comprised of 4 convolutional layers, with the number of filters set to [16, 32, 64, 32], followed by two fully connected layers with hidden units as 512 and 100. All convolutional layers used the kernel size (3, 3) and stride 2. The decoder consisted of two fully connected layers with 512 and 6272 hidden units followed by 4 transposed convolutional layers with channels [64, 32, 16, 1] respectively. ReLU non-linear activation was applied after every layer except for the last layer. We trained the models using the Adam^63^ optimizer for 150 epochs with an initial learning rate of $1e-3$ and decreased it by factors 2, 5, 10 after 30, 50 and 100 epochs respectively. The three loss functions were assigned the weights [1, 0.5, 0] for the first 20 epochs and subsequently changed to [1, 0.1, 1] respectively until convergence.

### Predictive model design using LbC

While conventional metrics such as cross entropy (for categorical-valued outputs) and mean squared error (for continuous-valued outputs) are commonly used, it has been recently found that interval calibration is effective for obtaining accurate and well-calibrated predictive models^28^. Hence, in TraCE, we adapt the Learn-by-Calibrating approach to train classifier (or regression) models that map from the CXR latent space to a desired target variable. By design, LbC provides prediction intervals in lieu of point estimates for the response $\text {y}$, i.e., $[\hat{{\text {y}}} - {{\updelta }}, \hat{{\text {y}}} + {{\updelta }}]$. Here, $\delta$ is used to define the interval. Suppose that the likelihood for the true response $\text {y}$ to be contained in the prediction interval is $p(\hat{{\text {y}}} - {{\updelta }} \le {\text {y}} \le \hat{{\text {y}}} + {{\updelta }})$, the intervals are considered to be well-calibrated if the likelihood matches the expected confidence level. For a confidence level $\alpha$, we expect the interval to contain the true response for $100\times \alpha \%$ of realizations from $p({\text {x}})$.

#### Algorithm

The model is comprised of two modules $\text {F}$ and $\text {G}$, implemented as neural networks, to produce estimates $\hat{{\text {y}}} = {\text {F}}({\text {z}})$ and ${\delta } = {\text {G}}({\text {z}})$ respectively. For example, in the case of multi-class classification settings, $\hat{{\text {y}}} \in {\mathbb {R}}^K$ is a vector of predicted logits for the *K* different classes. Since interval calibration is defined for continuous-valued targets, we adapt the loss function for training on the logits directly. To this end, we first transform the ground truth labels into logits. Note, for each sample, we allow a small non-zero probability (say 0.01) to all negative classes. As discussed earlier, suppose that the likelihood for the true ${\text {y}}[k], k \in (1,\ldots , K)$ to be contained in the interval is $p(\hat{{\text {y}}}[k] - \delta [k] \le {\text {y}}[k] \le \hat{{\text {y}}}[k] + \delta [k])$, the intervals are considered to be well-calibrated if the likelihood matches the confidence level. Denoting the parameters of the models $\text {F}$ and $\text {G}$ by ${\theta }$ and ${\phi }$ respectively, we use an alternating optimization strategy similar to^28^. In order to update ${\phi }$, we use the empirical interval calibration error as the objective:

8 $$\begin{aligned} {{\phi }}^* = \arg \min _{{{\phi }}}&\sum _{k=1}^K \left| \alpha - \frac{1}{N} \sum _{i=1}^N \mathbbm {1}\left[ (\hat{{\text {y}}}_i[k] - {\delta }_i[k]) \le {\text {y}}_i[k] \le (\hat{{\text {y}}}_i[k] + {\delta }_i[k])\right] \right| , \end{aligned}$$

where ${\delta }_i = {\text {G}}({\text {z}}_i;{{\phi }})$, and the desired confidence level $\alpha$ (set to 0.9 in our experiments) is an input to the algorithm. When updating the parameters ${\phi }$, we assume that the estimator ${\text {F}}(.;{ {\theta }})$ is known and fixed. Now, given the updated ${\phi }$, we learn the parameters ${\theta }$ using the following hinge-loss objective:

9 $$\begin{aligned} {{\theta }}^*&= \arg \min _{{{\theta }}} \sum _{k=1}^K \frac{1}{N} \sum _{i = 1}^N \left[ \max \left( 0, (\hat{{\text {y}}}_i[k] - {\delta }_i[k]) - {\text {y}}_i[k] +\tau \right) \right. \nonumber \\&\left. \quad +\max \left( 0, {\text {y}}_i[k] - (\hat{{\text {y}}}_i[k] + {\delta }_i[k]) +\tau \right) \right] , \end{aligned}$$

where $\hat{{\text {y}}}_i = {\text {F}}({\text {z}}_i; {{\theta }})$ and $\tau$ is the margin parameter (set to 0.05 in our experiments). Intuitively, for a fixed ${\phi }$, obtaining improved estimates for $\hat{{\text {y}}}$ can increase the empirical calibration error in (8) by achieving higher likelihoods even for lower confidence levels. However, in the subsequent step of updating ${\phi }$, we expect ${\delta }$’s to become sharper in order to reduce the calibration error. We repeat the two steps (Eqs. 8 and 9) until convergence.

### Architecture

As showed in Fig. 7b, the predictor was designed as a 7-layer fully connected network with hidden sizes [512, 1024, 256, 128, 64, 16, *K*] and ELU activations, while the interval estimator was a 6-layer network with sizes [512, 1024, 256, 128, 64, *K*] and ReLU activations.

### Uncertainty-aware counterfactual generation

CE modifies the counterfactual generation process in Eq. (1) using the pre-trained predictor and interval estimator models from LbC. Our goal is to generate explanations to support a given hypothesis on the target variable—for example emulating high-confidence disease states given the CXR of a healthy subject. To this end, we first obtain the latent representation for the given query image $\text {x}$ using the encoder, $\text {z} = {\text {E}}({\text {x}})$. We then use the pre-trained predictor ($\text {F}$) and interval estimator ($\text {G}$) models to generate the counterfactual $\bar{{\text {z}}}$. Finally, the generated counterfactuals in the latent space are passed to the decoder to obtain a reconstruction in the image space, $\bar{{\text {x}}}= {\text {D}}(\bar{{\text {z}}})$. We propose the following optimization to generate the counterfactual explanations:

10 $$\begin{aligned} \bar{{\text {z}}} = \arg \min _{\hat{{\text {z}}}} \eta _1 \Vert \text {z} - \hat{{\text {z}}}\Vert _2^2 + \eta _2 {{\mathcal {L}}}(\hat{{\text {y}}},\delta , \bar{{\text {y}}}) + \eta _3\delta , {\text { where }} \hat{{\text {y}}} = {\text {F}}(\hat{{\text {z}}}), \delta = {\text {G}}(\hat{{\text {z}}}) \end{aligned}$$

here, $\bar{{\text {y}}}$ is the desired value for the target attribute (hypothesis), $\eta _1, \eta _2, \eta _3$ are hyper-parameters for weighting the different terms, and $\Vert .\Vert _2$ denotes the $\ell _2$ norm. The first term ensures that the generated counterfactual is in the vicinity of the query sample $\text {x}$ (in the latent space). The second term ensures that the expected target value is contained in the prediction interval (calibration), while the final term penalizes arbitrarily large intervals to avoid trivial solutions. The calibration objective ${\mathcal {L}}$ is implemented as a hinge-loss term:

11 $$\begin{aligned} {\mathcal {L}}(\hat{{\text {y}}},\delta , \bar{{\text {y}}}) = \left[ \max \left( 0, (\hat{{\text {y}}} - \delta ) - \bar{{\text {y}}} +\tau \right) + \max \left( 0, \bar{{\text {y}}} - (\hat{{\text {y}}} + \delta ) +\tau \right) \right] , \end{aligned}$$

where the margin was fixed at $\tau =0.05$. Choosing $\eta _1, \eta _2, \eta _3$ is essential to controlling the discrepancy between $\text {z}$ and $\bar{{\text {z}}}$, and ensuring that the prediction for the counterfactual is $\bar{{\text {y}}}$.

### Baselines

 We considered a suite of baseline approaches for our empirical study and they differ by the strategies used for training the classifier, and counterfactual optimization. In particular, we investigate approaches that produce explicit uncertainty estimators as well as those that directly build well-calibrated predictors. However, note that, all methods perform their optimization in the same latent space.

1. *Vanilla* In this approach, we train the classifier with no explicit calibration or uncertainty estimation, and use the following formulation to generate the counterfactuals:
  12 $$\begin{aligned} \bar{{\text {z}}} = \arg \min _{\hat{{\text {z}}}} \eta _1 \Vert {\text {z}} - \hat{{\text {z}}}\Vert _2^2 + \eta _2 {\mathcal {L}}_{ce}\left[ {\text {F}}(\hat{{\text {z}}}), \bar{{\text {y}}}\right] , \end{aligned}$$
  where ${\mathcal {L}}_{ce}$ denotes the cross entropy loss.
2. *Mixup* This is a popular augmentation strategy^64^ that convexly combines random pairs of images and their labels, in order to temper overconfidence in predictions. Recently, in Ref.^65^, it was found that mixup regularization led to improved calibration in the resulting model. In mixup, the model is trained not only on the training data, but also using samples in the vicinity of each training sample:
  13 $$\begin{aligned} {\text {x}} = \lambda {\text {x}}_i + (1-\lambda ) {\text {x}}_j; \quad {\text {y}} = \lambda {\text {y}}_i + (1-\lambda ) {\text {y}}_j, \end{aligned}$$
  where ${\text {x}}_i$ and ${\text {x}}_j$ are randomly chosen samples with labels ${\text {y}}_i$ and ${\text {y}}_j$. The parameter $\lambda$, drawn from a symmetric Beta distribution, determines the mixing ratio. Since this approach does not produce any uncertainty estimation, the counterfactual optimization is same as that of the *Vanilla* approach in Eq. (12).
3. *MC dropout* In this baseline, we train the classifier with dropout regularization and estimate the (epistemic) prediction uncertainty for any test sample by running multiple forward passes. Finally, we use the following heteroscedastic regression objective to implement uncertainty-based calibration during counterfactual optimization:
  14 $$\begin{aligned} \bar{{\text {z}}} = \arg \min _{\hat{{\text {z}}}} \eta _1 \Vert {\text {z}} - \hat{{\text {z}}}\Vert _2^2 + \eta _2 \left[ \frac{(\bar{{\text {y}}} - \mu _{\hat{{\text {z}}}})^2}{2 \sigma _{\hat{{\text {z}}}}^2} + \frac{1}{2} \log (\sigma _{\hat{{\text {z}}}}^2) \right] . \end{aligned}$$
  Note, similar to the proposed approach, here we operate directly on the logits and the mean/variance estimates ($\mu _{\hat{{\text {z}}}}, \sigma _{\hat{{\text {z}}}}^2$) are obtained using *T* (set to 5) forward passes with dropout.
4. *Deep ensembles* Deep ensembles form an important class of uncertainty estimation methods, wherein the model variance is used as a proxy for uncertainties. In this approach, we independently train *M* different models (with bootstrapping and different model initializations) with the same architecture. Subsequently, for any input sample $\text {x}$, we obtain the mean/variance estimates ($\mu _{\hat{{\text {z}}}}, \sigma _{\hat{{\text {z}}}}^2$) by aggregating predictions from the *M* models. Finally, we employ the calibration objective in Eq. (14) to perform counterfactual optimization. While highly accurate and currently one of the best uncertainty estimation techniques, deep ensembles require training multiple models, which can become a computational bottleneck when training deep networks.
5. *Uncertainty-Weighted confidence calibration (UWCC)* The authors in Ref.^49^ proposed to build calibrated classification models by augmenting a confidence-calibration term to the standard cross-entropy loss and weighting the two terms using the uncertainty measured via multiple stochastic inferences. Mathematically,
  15 $$\begin{aligned} \frac{1}{N}\sum _{i=1}^N -(1 - \alpha _i)\log (P(\hat{{\text {y}}}_i|{\text {z}}_i)) + \alpha _i D_{KL}({\text {U}}({\text {y}})||P(\hat{{\text {y}}}_i|{\text {z}}_i)) \end{aligned}$$
  here the first term denotes the cross-entropy loss, and the predictions $P(\hat{{\text {y}}}_i|{\text {z}}_i)$ are inferred using stochastic inferences for ${\text {z}}_i$, while the variance ($\alpha _i$) in the predictions is used to balance the loss terms. More specifically, we perform *T* forward passes with dropout in the network and promote the softmax probabilities to be closer to an uniform distribution, i.e. high uncertainty, when the variance is large. The normalized variance $\alpha _i$ is given by the mean of the Bhattacharyya coefficients between each of the *T* predictions and the mean prediction. Since the model is inherently calibrated during training, we do not measure the uncertainties at test time and hence use the optimization in Eq. (12) for generating counterfactuals. For the case of continuous-valued targets (i.e., regression tasks), we utilize the extension in Ref.^66^ that performs heteroscedastic calibration of the MC dropout estimator during training.

## Data Availability

All datasets used in this were obtained from publicly released databases and pre-processed using open-source tool chains. We have added appropriate links to obtain the data as well as access the scripts for pre-processing, wherever applicable.

## Code availability

The software associated with this paper will be hosted through a public code repository (github).
